# Reanalysis of the Rituximab in ANCA-Associated Vasculitis trial identifies granulocyte subsets as a novel early marker of successful treatment

**DOI:** 10.1186/s13075-015-0778-z

**Published:** 2015-09-21

**Authors:** Mazen Nasrallah, Yannick Pouliot, Bjoern Hartmann, Patrick Dunn, Elizabeth Thomson, Jeffrey Wiser, Atul J. Butte

**Affiliations:** Institute for Computational Health Sciences, University of California, San Francisco, 550 16th Street, Box 0110, Mission Hall 4733, San Francisco, CA 94158 USA; Department of Medicine, Cleveland Clinic Foundation, Cleveland, OH 44106 USA; Division of Systems Medicine, Department of Pediatrics, Stanford University School of Medicine, Stanford, CA 94305 USA; Division of Immunology and Rheumatology, Department of Medicine, Stanford University School of Medicine, Stanford, CA 94305 USA; Northrop Grumman Information Systems Health IT, Rockville, MD 20850 USA

## Abstract

**Introduction:**

In the present study, we sought to identify markers in patients with anti-neutrophil cytoplasmic antibody (ANCA)-associated vasculitis (AAV) that distinguish those achieving remission at 6 months following rituximab or cyclophosphamide treatment from those for whom treatment failed in the Rituximab in ANCA-Associated Vasculitis (RAVE) trial.

**Methods:**

Clinical and flow cytometry data from the RAVE trial were downloaded from the Immunology Database and Analysis Portal and Immune Tolerance Network TrialShare public repositories. Flow cytometry data were analyzed using validated automated gating and joined with clinical data. Lymphocyte and granulocyte populations were measured in patients who achieved or failed to achieve remission.

**Results:**

There was no difference in lymphocyte subsets and treatment outcome with either treatment. We defined a Granularity Index (GI) that measures the difference between the percentage of hypergranular and hypogranular granulocytes. We found that rituximab-treated patients who achieved remission had a significantly higher GI at baseline than those who did not (*p* = 0.0085) and that this pattern was reversed in cyclophosphamide-treated patients (*p* = 0.037). We defined optimal cutoff values of the GI using the Youden index. Cyclophosphamide was superior to rituximab in inducing remission in patients with GI below −9.25 % (67 % vs. 30 %, respectively; *p* = 0.033), whereas rituximab was superior to cyclophosphamide for patients with GI greater than 47.6 % (83 % vs. 33 %, respectively; *p* = 0.0002).

**Conclusions:**

We identified distinct subsets of granulocytes found at baseline in patients with AAV that predicted whether they were more likely to achieve remission with cyclophosphamide or rituximab. Profiling patients on the basis of the GI may lead to more successful trials and therapeutic courses in AAV.

**Trial registration:**

ClinicalTrials.gov identifier (for original study from which data were obtained): NCT00104299. Date of registration: 24 February 2005.

**Electronic supplementary material:**

The online version of this article (doi:10.1186/s13075-015-0778-z) contains supplementary material, which is available to authorized users.

## Introduction

Granulomatosis with polyangiitis (GPA) and microscopic polyangiitis (MPA) are severe, necrotizing small-vessel vasculitides. These diseases are classified as anti-neutrophil cytoplasmic antibody–associated vasculitis (AAV) on the basis of their association with anti-neutrophil cytoplasmic antibodies (ANCA) that target proteinase 3 (PR3) or myeloperoxidase (MPO) antigens [1]. Until recently, cyclophosphamide and glucocorticoids were the mainstays for the induction of remission. However, the Rituximab in ANCA-Associated Vasculitis (RAVE) trial (ClinicalTrials.gov identifier: NCT00104299) clearly demonstrated the non-inferiority of rituximab to cyclophosphamide for the induction of remission [2].

RAVE was a prospective, randomized, active-controlled, double-blinded, double-dummy, phase II/III, multicenter interventional study involving 197 participants in which rituximab was compared with cyclophosphamide for the induction of remission in AAV. Its primary endpoint was the induction of complete remission, defined as a disease score of zero and a complete tapering off from steroids. RAVE’s primary results demonstrated that rituximab was non-inferior to cyclophosphamide for the induction of complete remission and was more efficacious than cyclophosphamide for the induction of remission in patients with relapsing disease [2].

Nonetheless, approximately 35 % of patients treated with rituximab and 47 % of patients treated with cyclophosphamide failed to achieve complete remission, with no clinical or biochemical markers identified so far that correlate with treatment outcome [2, 3]. We aimed to address this lacuna by applying computational approaches to reanalyze publicly available data from the first 6 months of the RAVE trial. These data were obtained from two publicly accessible sources: Immunology Database and Analysis Portal (ImmPort; import.niaid.nih.gov or immport.org; accession number SDY91) [4] and the Immune Tolerance Network’s (ITN) TrialShare ([5]; accession number ITN021AI).

We hypothesized that patients who achieved complete remission on either rituximab or cyclophosphamide had differences in their white blood cell compartment detectable at the beginning of their treatment course, compared with those who failed remission. To test our hypothesis, we reanalyzed 1150 flow cytometry sample measurements using the automated-gating software tool Flow cytometry Clustering without K (ImmPort-FLOCK) [6], available online at the ImmPort website (immport.niaid.nih.gov). We developed and validated a relational database implementation of the FLOCK-generated automated-gating results for rapid integration with the trial’s other mechanistic and clinical data. Using this approach, we were able to compare the percentages of total lymphocytes, lymphocyte subsets, and granulocyte subsets in patients with AAV who met the primary endpoint outcome of the RAVE trial with those who did not.

## Methods

### Study design and data sources

Subjects in this study were enrolled in the RAVE trial (ClinicalTrials.gov identifier: NCT00104299) [2]. Deidentified clinical and partial mechanistic data from the first 6 months of the RAVE trial are publicly available and were used in the present study. As part of the trial, investigators ran flow cytometry assays on whole-blood specimens from 197 study participants. We downloaded these data, as well as clinical trial data, from the ITN TrialShare [5] from the first 6 months of the trial (the only time period for which the data were available). This yielded a total of 1150 FCS-formatted files and 22 clinical data files. Both clinical trial data and flow cytometry data are now also available from the National Institute of Allergy and Infectious Diseases (NIAID) ImmPort database [7]. Regarding the reanalysis of such open clinical trials data, we did not have to contact the original trialists for this study; questions regarding the RAVE study design were addressed by review of the primary publications from the RAVE trial. Technical questions regarding the clinical or mechanistic data files were directed to the study coordinator at the ITN TrialShare Network.

### Description of the flow cytometry files

The RAVE trial serially collected whole blood that was assayed by flow cytometry using standardized protocols. For each patient, blood was collected at an initial screening visit, as well as at week 2 and months 1, 2, 4, 6, 9, 12, 15 and 18 following enrollment [8]. Per protocol, a total of 15 antibody-staining panels were assessed by flow cytometry on each sample. Only one panel, assessing the B1 B-cell population through surface protein expression of cluster of differentiation 1c (CD1c), CD5, CD19, CD21 or CD23, was publicly available at the time of our study and was downloaded.

### ImmPort-FLOCK analysis of flow cytometry data files

FCS files were uploaded and analyzed using the ImmPort web-based FLOCK service (ImmPort-FLOCK) [9]. We chose ImmPort-FLOCK over other auto-gating algorithms because it is relatively robust to intersample fluorescence variability [6] and because the algorithm performed well in comparison with other auto-gating algorithms [10]. Version 1 of ImmPort-FLOCK, previously validated in the analysis of small-volume flow cytometry data [6, 11, 12], was used for our analyses with default settings. The analysis results were visualized using the ImmPort-FLOCK data visualization tool (Additional file 1, panel a).

We then downloaded the results for each analyzed FCS file and processed them for upload into a relational database (MySQL; Oracle, Redwood City, CA, USA). The database also contained the clinical data, enabling joining with the ImmPort-FLOCK results. SQL queries were used to extract flow cytometry results as described below.

### Using ImmPort-FLOCK database to identify cell populations

A description of the automated gating in the ImmPort-FLOCK algorithm was previously published [6]. Each cell population identified by ImmPort-FLOCK is defined by parameters measured by the flow cytometer: forward scatter (FSC), side scatter (SSC), and fluorescence signal intensity. An example of ImmPort-FLOCK output is shown in Additional file 1, panel a. Each cell population (cluster) identified by ImmPort-FLOCK is color-coded and represented by a vector of signal values on a scale of 1–4, along with the corresponding percentage of cells in a cluster relative to the total cell population. The signal values represent a scale that qualitatively describes expression levels: 1 = negative, 2 = low, 3 = positive, and 4 = high. For example, a population of cells with the values FSC = 2, SSC = 1, CD19 = 3, CD21 = 2, CD23 = 2, CD5 = 2 and CD1c = 1 would be small (FSC = 2); agranular (SSC = 1); positive for CD19; weakly positive for CD21, CD23 and CD5; and negative for CD1c. These values are normalized within each file, allowing comparison across different samples. Additional file 1, panel b, is a summary of expression profiles for each population in a typical flow cytometry file, with a total of 21 populations identified by ImmPort-FLOCK. Auto-gating results from all 1150 FCS files were stored in a relational database and linked to each subject’s clinical data. The database was then queried to identify cell counts within specific populations. Additional file 1, panel c, shows an FSC-SSC dot plot to show how each population was defined on the basis of size and granularity. Table 1 shows the queries we used to identify the different populations. To validate our method, 100 FCS files were randomly chosen from the total set and analyzed manually by two independent immunologists using the software package FlowJo (Tree Star, Ashland, OR, USA) and compared with ImmPort-FLOCK results.

**Table 1:** Queries used to identify different cell populations using our database storing results from ImmPort-FLOCK

Cell populations	Queries used
Total lymphocytes	FSC ≤ 2 AND SSC = 1
CD1c + lymphocytes	FSC ≤ 2 AND SSC = 1 AND CD1c ≥ 2
CD5+ lymphocytes	FSC ≤ 2 AND SSC = 1 AND CD5 ≥ 2
CD19+ lymphocytes	FSC ≤ 2 AND SSC = 1 AND CD19 ≥ 2
CD21+ lymphocytes	FSC ≤ 2 AND SSC = 1 AND CD21 ≥ 2
CD23+ lymphocytes	FSC ≤ 2 AND SSC = 1 AND CD23 ≥ 2
Total monocytes	FSC ≥ 3 AND SSC ≤ 2
Total granulocytes	((FSC ≥ 3 AND SSC ≥ 3) OR (FSC ≤ 2 AND SSC ≥ 2))
Hypergranular granulocytes	FSC ≥ 1 AND SSC = 4
Hypogranular granulocytes	(FSC ≤ 2 AND SSC = 3) OR (FSC ≤ 2 AND SSC = 2) OR (FSC ≥ 3 AND SSC = 3)

*CD* cluster of differentiation, *FSC* forward scatter, ImmPort-FLOCK, Immunology Database and Analysis Portal flow cytometry clustering without K, *SSC* side scatter

### Granularity Index

The size and granularity of cells are measured indirectly by FSC and SSC signals, respectively. Within the granulocyte population, we defined two populations of granulocytes on the basis of granularity: cells with high granularity (Fig. 3a), which we call *hypergranular granulocytes*, and cells with lower granularity, which we term *hypogranular granulocytes*. We calculate a GI by taking the absolute difference between the percentages of these two populations, potentially ranging from −100 % to +100 %. For example, if hypergranular granulocytes constitute 40 % of the total white blood cell population and hypogranular granulocytes constitute 20 % the total white blood cell population, then the Granularity Index (GI) is 20 %. The specific cell population query definitions we used for these two populations are shown in Table 1.

### Statistical analysis

Statistical analyses were performed using the R statistical software package. Two-sample comparisons were performed with the use of an unpaired Kruskal-Wallis rank-sum non-parametric test for continuous measures and a two-tailed Fisher’s exact test for binary measures. *R*^2^ correlation values were calculated using a linear regression model with Pearson correlation coefficients. Null hypotheses were rejected at the 0.05 significance level.

## Results

### Analytical process

**Fig. 1:**
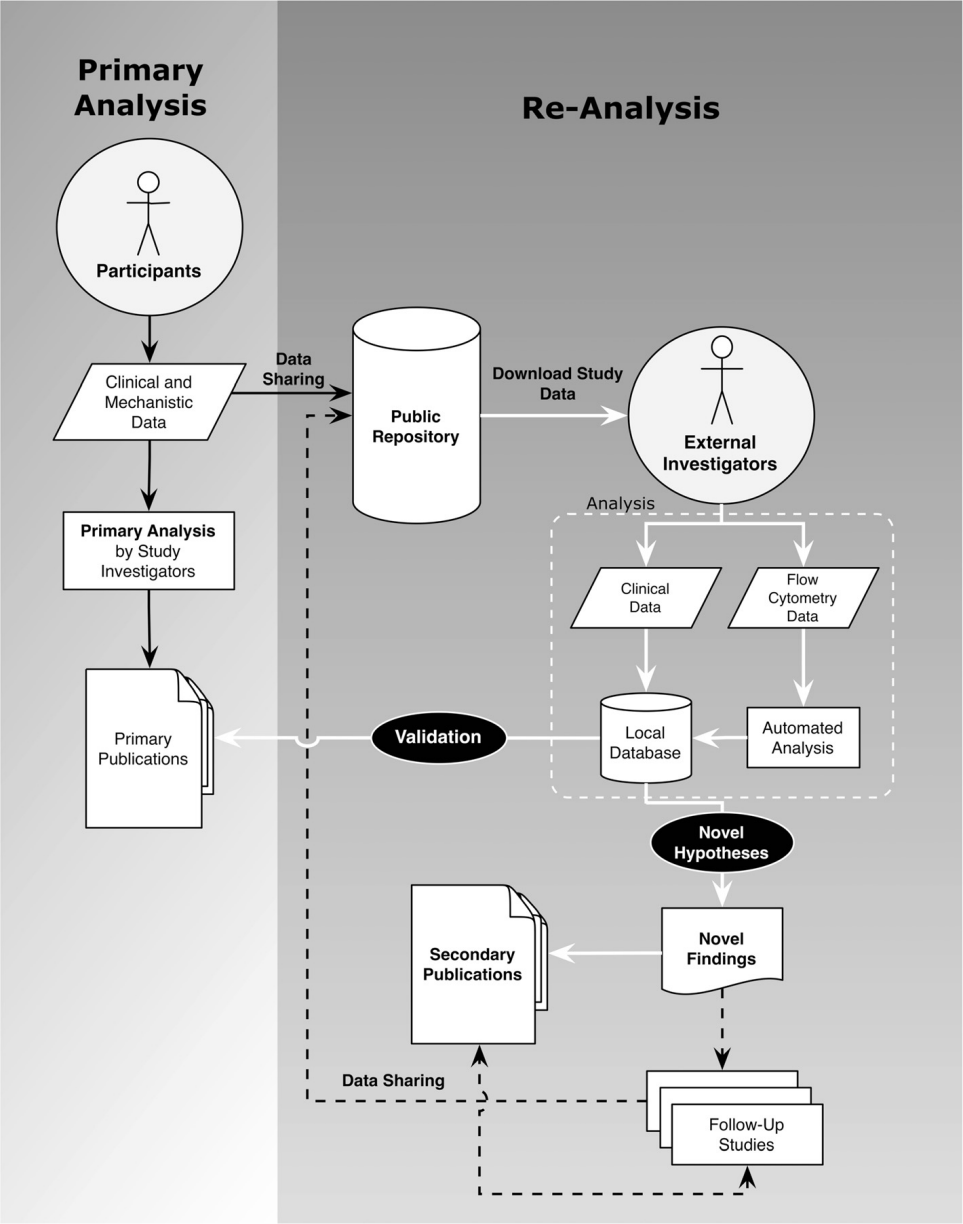
Overview of the analytical process, starting with open access clinical trial data. *Solid black arrows* represent work carried out by the primary investigators. *White arrows* represent work done in the present study, enabled by the public accessibility of the original raw trial data. *Dashed black arrows* represent future work that could be triggered by the reanalysis process

Figure 1 shows an overview of our analytical process. Publicly available, raw, deidentified, individual-level data from the RAVE trial were obtained from ITN TrialShare and ImmPort. These data covered 197 individuals studied over 6 months. Whole blood had been obtained from these individuals during the RAVE study, and cell populations were analyzed by flow cytometry using standardized protocols. A total of 1150 flow cytometry files were reanalyzed using the automated gating tool ImmPort-FLOCK, and the results were stored in a database. We used this database to execute structured queries to retrieve flow cytometry associated with clinical data.

### Validation of the ImmPort-FLOCK identified cell populations and database

**Fig. 2:**
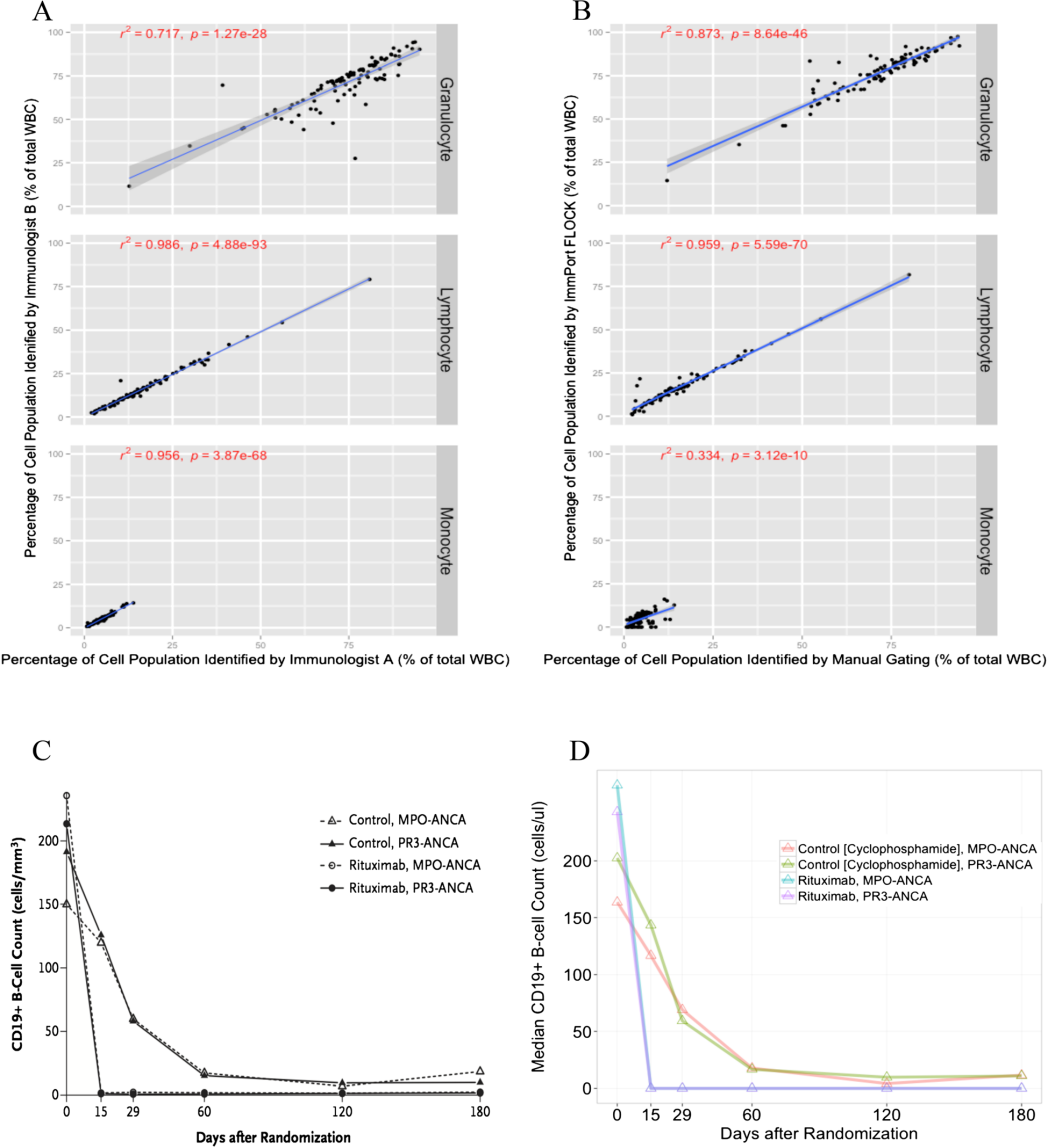
Validation of the Immunology Database and Analysis Portal flow cytometry clustering without K (ImmPort-FLOCK). Cell subset percentages by automated identification were validated against manual gating for the identification of immune cell populations on the basis of size and granularity (forward scatter and side scatter, respectively). One hundred random flow cytometry files were independently analyzed by two immunologists using standard FlowJo software. **a** Scatterplots between the two immunologists show significant concordance in the identification of different cell populations. **b** Similar correlation was seen for granulocyte and lymphocyte percentages between automated analysis and the average of the two immunologists (shaded area represents the 95 % confidence interval of the regression line; *p* values based on Pearson correlation test). **c** Originally published figure showing the drop in CD19+ B-cell counts with rituximab or control (cyclophosphamide) treatment generated using manual gating of flow cytometry results (Reproduced with permission from [2]. **d** Results obtained through automated identification of the CD19+ lymphocyte population. Results shown in (c) and (d) represent median cell counts. *ANCA* anti-neutrophil cytoplasmic antibodies, *CD* cluster of differentiation, *MPO* myeloperoxidase, *PR3* proteinase 3

We first validated our approach for extracting cell populations by comparing ImmPort-FLOCK auto-gating results with the current gold standard of manual gating of flow cytometry data. The results of this validation are shown in Fig. 2. We found that cell percentages identified through automated gating correlated well with results from manual gating obtained from two immunologists identifying lymphocytes and granulocytes (r^2^ = 0.959 and 0.873, respectively). Correlation was lower for automated identification of monocytes: r^2^ = 0.334. The inter-rater correlation between the two immunologists was also very good for lymphocytes, monocytes and granulocytes (r^2^ = 0.986, r^2^ = 0.956 and r^2^ = 0.717, respectively). We also validated our approach against published cell counts from the RAVE trial that showed a drop in the absolute CD19+ lymphocyte counts. ImmPort-FLOCK results were highly congruent with the published RAVE results obtained by manual analysis (Fig. 2c and d) with an r^2^ of 0.99 (Additional file 2).

### Patient characteristics at screening

Of the 197 study participants enrolled in the RAVE trial, 187 patients had flow cytometry measurements obtained from samples at their screening visit, before treatment. These 187 patients included 94 male and 93 female patients with a mean age of 52.9 years. All patients had severe disease at baseline (mean Birmingham Vasculitis Activity Score for Wegener’s granulomatosis 8, range 3–23). Diagnoses comprised 137 with GPA, 48 with MPA, and 1 with indeterminate disease, and 1 had a missing diagnosis. Of the 187 patients, 123 were positive for anti-PR3, 64 were positive for anti-MPO antibodies, 93 were randomized to cyclophosphamide treatment and 94 were randomized to receive rituximab.

The primary endpoint of the RAVE trial was the induction of complete remission, defined as a disease score of zero and a complete tapering off from steroids. After 6 months in the trial, 48 (52 %) of 93 in the cyclophosphamide group reached the primary endpoint, compared with 60 (64 %) of 94 in the rituximab group. In congruence with previously published data from the RAVE trial, we did not identify clinical variables able to discriminate between those who met the primary endpoint outcome and those who did not (Table 2).

**Table 2:** Baseline characteristics of subjects treated with either rituximab or cyclophosphamide and stratified by primary endpoint outcome

	Rituximab		*p* Value	Cyclophosphamide		*p* Value
	Success, *n* = 60	Failure, *n* = 34		Success, *n* = 48	Failure, *n* = 45	
Age	53.25	56	0.44	51.3	51.9	0.85
Sex			0.52			0.68
Male	45 %	53 %		50 %	56 %	
Female	55 %	47 %		50 %	44 %	
ANCA-associated vasculitis type			0.66			0.27
PR3	67 %	62 %		60 %	66 %	
MPO	33 %	38 %		40 %	34 %	
Newly diagnosed at enrollment	48 %	50 %	1.0	56 %	36 %	0.06
BVAS/WG	7.97 ± 0.36	8.09 ± 0.52	0.85	8.42 ± 0.53	7.60 ± 0.47	0.25

*ANCA* anti-neutrophil cytoplasmic antibodies, *BVAS-WG* Birmingham Vasculitis Activity Score for Wegener’s granulomatosis, *MPO* myeloperoxidase, *PR3* proteinase 3

### Overview of population changes with treatment outcome

We hypothesized that patients who achieved complete remission by month 6 in either arm of the trial had differential changes at baseline (i.e., before the initiation of treatment) in their leukocyte composition compared with those who did not. We examined the percentage of major lymphocyte subpopulations as well as the percentage of granulocytes. There was no difference in the percentage of major lymphocyte subsets at baseline (CD1c^+^, CD5^+^, CD19^+^, CD21^+^ or CD23^+^ lymphocytes) between patients who achieved or failed to achieve complete remission on either rituximab or cyclophosphamide (data not shown).

### Distinct granulocyte populations at baseline are associated with treatment outcome

**Fig. 3:**
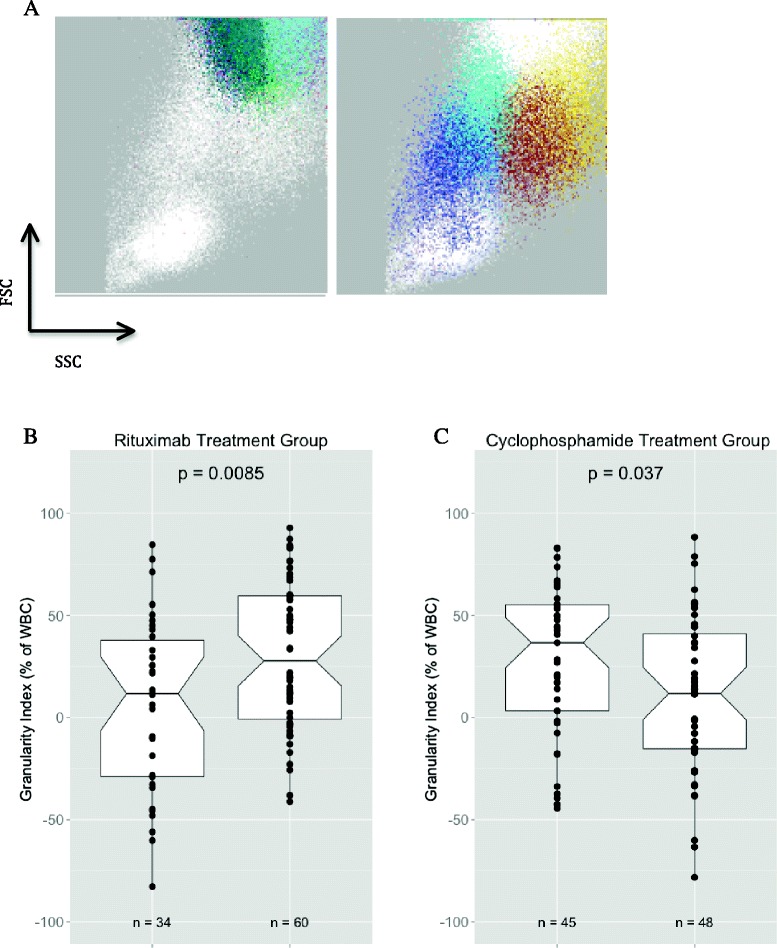
Granulocyte subpopulations and treatment outcomes. **a** Representative bidimensional dot plot illustrating the granulocyte subpopulations identified in an automated manner. *Left*: Hypergranular granulocytes (definitions in Table 1). *Right*: Hypogranular granulocytes. *FSC* forward scatter, *SSC* side scatter. **b** and **c** Granularity Index at day 0 among patients receiving rituximab and cyclophosphamide, respectively, stratified by treatment outcome. Data distribution is shown as a notched boxplot (showing minimum, maximum, 25th percentile, median and 75th percentile). An unpaired Kruskal-Wallis rank-sum non-parametric test was used to calculate significance. *WBC* white blood cells

SSC signals can be used as a rough semiquantitative measure of granulocyte granularity and primary granule secretory responses, thus providing information on cell activation status [13]. Using ImmPort-FLOCK, we identified distinct granulocyte subsets on the basis of size and granularity and calculated a GI as described in the Methods section. We assigned this index to each individual at baseline. We found that on day 0, the GI was higher in the 60 rituximab-treated patients who achieved complete remission than in the 34 patients who did not (*p* = 0.0085) (Fig. 3b). In juxtaposition, the GI was lower in the 48 cyclophosphamide-treated patients who achieved complete remission than in the 45 patients who did not (*p* = 0.037) (Fig. 3c).

We found no association between the GI at baseline and baseline ANCA titers (*r*^2^ = 0.0016), disease severity score (*r*^2^ = 0.001), age (*r*^2^ = 0.0056), sex (*p* = 0.46) or relapsing disease at baseline (*p* = 0.10). However, individuals with anti-PR3 ANCA vasculitis had significantly higher GI compared with those with anti-MPO disease (*p* = 0.017). As shown in Table 2, there was no difference in the attainment of primary endpoint outcome and the serological subtype (anti-PR3 vs. anti-MPO disease).

Next, we calculated the optimal cutoff of GI values using the Youden index [14] to discriminate between those who achieved or failed to achieve the primary endpoint outcome. The optimal cutoff for identifying patients failing rituximab treatment was calculated as −9.25 %. Fourteen (41.2 %) of the thirty-four patients who failed to achieve remission on rituximab had a GI below this cutoff value, compared with only six (10 %) of sixty patients who achieved remission (*p* = 0.0011, likelihood ratio [LR] 4.12, 95% confidence interval [CI] 1.74–9.72). Of note, lower GI values correlated with higher likelihood of failure to rituximab. For example, only 2 (3.3 %) of 60 patients who achieved complete remission on rituximab had a GI below −27 %, compared with 10 (29.4 %) of 34 patients who responded (*p* = 0.0005, LR = 8.82, CI 2.05–37.95), whereas none of the responders had a GI below −43 %, compared with 18 % of non-responders (*p* = 0.0017). The optimal threshold GI for identifying patients achieving complete remission on rituximab was calculated at 47.6 %. Twenty-five (41.6 %) of the sixty patients who achieved remission on rituximab had a GI above this cutoff, compared with only five (14.7 %) of thirty-four patients who failed to achieve remission (*p* = 0.01, LR 2.83, CI 1.20–6.72).

We then applied these threshold GI values to the group of patients treated with cyclophosphamide. Among the 48 patients who achieved complete remission on cyclophosphamide, 16 (33 %) had a GI below −9.25 % compared with only 8 (17.8 %) of 45 patients who failed to achieve complete remission, although this difference was not significant (*p* = 0.10). Conversely, 18 (40.0 %) of 45 patients who failed to achieve complete remission had a GI above 47.6 % compared with only 9 (18.8 %) of 48 patients who achieved complete remission (*p* = 0.0387, LR 2.13, CI 1.07–4.25).

**Fig. 4:**
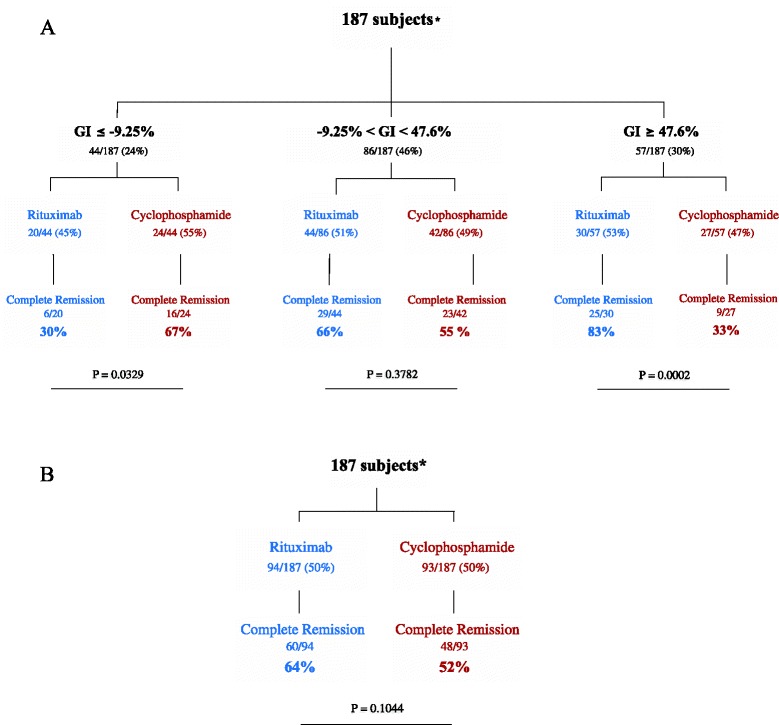
Complete remission rates for patients in the Rituximab in ANCA-Associated Vasculitis (RAVE) trial. **a** Primary endpoint outcomes among RAVE trial subjects stratified by the Granularity Index (GI). **b** Primary endpoint outcomes among RAVE trial subjects treated with either rituximab or cyclophosphamide in the absence of stratification. Fisher’s exact test was used to calculate significance between rates of complete remission on rituximab and cyclophosphamide. *10 of the 197 original trial subjects did not have flow cytometry data at baseline

Using these cutoff values, we define three subsets of patients, as shown in Fig. 4a: patients with a GI at or below −9.25 %, patients with a GI between −9.25 % and 47.6 %, and patients with a GI at or above 47.6 %. Of the 187 patients, 44 (24 %) had a GI below −9.25 %. Within this group, cyclophosphamide was superior to rituximab in inducing complete remission (67 % vs. 30 %, respectively; *p* = 0.033). Another 86 patients (46 %) had a GI between −9.25 % and 47.6 %. Within this group, the remission rate for patients treated with rituximab was 66 % compared with 55 % for those who received cyclophosphamide (*p* = 0.38), and it was also similar to the remission rate among patients randomly assigned to receive rituximab or cyclophosphamide (Fig. 4b). Finally, the remaining 57 patients (30 %) had a GI above 47.6 %, and, within this group, rituximab was significantly more effective than cyclophosphamide in inducing complete remission (83 % vs. 33 %, respectively; *p* = 0.0002).

## Discussion

We used publicly available flow cytometry data from the RAVE clinical trial to discover leukocyte markers that could distinguish patients benefiting from either studied treatment. Our reanalysis study is important for three reasons. First, we are highlighting the power of free, publicly accessible, high-quality raw clinical trial data available through NIAID ImmPort and the Immune Tolerance Network. Second, we evaluated and validated the use of automated gating for the analysis of bulk clinical trial flow cytometry data. Finally, we show that the reanalysis of existing data can generate new discoveries.

A vast amount of valuable clinical research data is generated as part of clinical trials; unfortunately, most of these data are never published [15]. Several measures are being taken in the hope of encouraging data sharing, such as the BMJ open data campaign and the AllTrials initiative [16–18]. We show that raw data released in a deidentified manner can confirm results achieved by the original investigators, indicating the fidelity of the data-sharing mechanism from investigator to public user. We believe this helps address concerns regarding data-sharing mechanisms raised with the recent calls for the release of raw individual-level clinical trial data [19, 20].

Given how much raw, publicly available flow cytometry measurement data were available for our study, we applied and validated an automated gating technique instead of manual gating, and reproduced previously published data. Although ImmPort-FLOCK has been used to analyze flow cytometry data for several years [6, 11, 12], to the best of our knowledge, auto-gating has not been evaluated in the analysis of large-volume flow cytometry data from clinical trials as a means of reducing the variability associated with human analysts. Our methodology of using ImmPort-FLOCK for mass analysis was very accurate in identifying the lymphocyte and granulocyte populations; however, it was less accurate in correctly identifying the rarer monocyte population. Accurate assessment of this population would require additional surface protein staining.

The reanalysis of publicly available molecular databases has previously been shown to lead to clinically relevant findings, including new oncogenes in cancer [21], new therapeutic targets for chronic diseases [22] and new uses for existing drugs [23]. Here, through the reanalysis of flow cytometry measurement data, we found that the differential composition of the leukocyte compartment at baseline was significantly associated with patients achieving a primary study endpoint in the RAVE trial.

We used SSC signals to show that patients with AAV who responded to rituximab had more hypergranular granulocytes, and fewer hypogranular granulocytes, at baseline than those who did not respond. Unexpectedly, this pattern was the opposite in patients receiving cyclophosphamide. It is tempting to speculate that distinct patient subgroups exist on the basis of granulocyte subsets alone and that they will have differential responses to rituximab and cyclophosphamide. Although these findings are novel, the role of neutrophils and ANCA in disease pathophysiology is well known. ANCA targeting primary granule antigens expressed on the neutrophils are known to elicit neutrophil activation and degranulation and subsequently mediate tissue damage [24–26]. After identifying an optimal cutoff value for our GI, we showed that individuals above or below this threshold were significantly more likely to either achieve or fail to achieve remission.

**Fig. 5:**
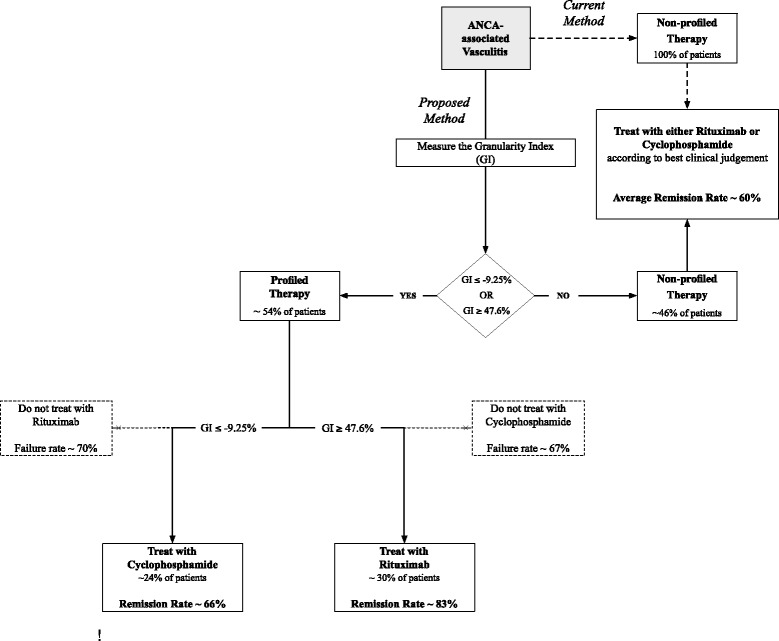
Proposed “personalized” treatment algorithm for anti-neutrophil cytoplasmic antibody (ANCA)-associated vasculitis on the basis of the Granularity Index

On the basis of these findings, we propose a novel method of profiling patients on the basis of the GI (Fig. 5). Under this schema, patients with AAV with a GI at or below −9.25 % or at or above 47.6 % (about 54 % of patients with AAV) would significantly benefit from “personalized” or profiled therapy with cyclophosphamide or rituximab, respectively. Patients with a GI between −9.25 % and 47.6 % (about 46 % of AAV patients) would be treated with either therapy according to best clinical judgment, similar to current treatment practice. We predict that if the RAVE trial were executed with this profiled design, the overall remission rate would jump from 58 % (108 of 187) to 69 % (129 of 187) (*p* = 0.0317).

Our study was limited by the availability of flow cytometry data from the RAVE trial. For example, the sole antibody panel released by the RAVE investigators did not specifically evaluate the granulocyte compartment, such that we were unable to further characterize these granulocytes. With the anticipated release of additional antibody panels and other mechanistic data from the RAVE trial, we believe we will be able to further characterize these patients’ granulocyte compartment and test our predictors. Our analysis was also limited by the small sample size, which precluded us from validating our model in a separate test sample. Hence, we acknowledge that the model may overfit the data and would require further validation in future studies.

Although we were unable to validate our findings in an independent cohort, a study by Grayson et al. published while this article was in peer review demonstrated the presence of a low-density granulocyte (LDG) gene expression signature in the population of RAVE trial patients who did not respond to treatment [27]. Whether this population of LDG corresponds to the hypogranular granulocyte population described in our present report is uncertain. However, we believe our results complement those of Grayson et al. and further demonstrate that certain patients might benefit from a more targeted therapy with either cyclophosphamide or rituximab on the basis of their GI.

Given our findings and the independent findings of Grayson et al., we believe it would be useful to further evaluate the granulocyte subset using targeted staining panels in future clinical studies. Indeed, flow cytometric analysis of peripheral blood tends to focus on the analysis of the lymphocyte compartment (and less so the mononuclear compartment) and generally ignores the granulocyte compartment. Routinely assessing this granulocyte compartment in future clinical studies may help elucidate their role not only in AAV but in other diseases as well.

## Conclusions

We identified, within the RAVE trial cohort, novel changes in granulocyte subpopulations that discriminate patients with AAV who achieved complete remission on either rituximab or cyclophosphamide from those who did not. Equally important, our study demonstrates the utility of data sharing and encourages further efforts to promote the dissemination of raw, individual-level clinical trial data. We hope our specific findings prompt follow-up studies that ultimately translate into improved quality of patient care.

## Additional files

Additional file 1:
**ImmPort-FLOCK Output.** Sample FLOCK analysis of an FCS file (903935_SN05_I019.fcs). Distinct populations identified by clustering are color-coded. a Dot plot representations of populations defined by FSC, SSC, CD1c, CD21, CD5, CD19 and CD23. b Summary of each population described by an expression profile of approximate marker intensity (1 = negative, 2 = low, 3 = positive, 4 = high). c Gridding definitions used to identify total lymphocyte, monocyte and granulocyte populations on the basis of FSC and SSC. (PDF 643 kb) Additional file 2:
**Correlation between CD19+ B cells identified manually versus via ImmPort-FLOCK.** RAVE flow cytometry files were analyzed using ImmPort-FLOCK, and the population of CD19+ lymphocytes was identified for each of the 1150 files. Publicly available manual gating results from primary RAVE trial investigators (*x*-axis) were compared with ImmPort-FLOCK results (*y*-axis). A regression coefficient was calculated using a linear regression model. (PDF 49 kb)

## Abbreviations

AAV: anti-neutrophil cytoplasmic antibody–associated vasculitis
ANCA: anti-neutrophil cytoplasmic antibodies
BVAS-WG: Birmingham Vasculitis Activity Score for Wegener’s granulomatosis
CD: cluster of differentiation
CI: 95 % confidence interval
FLOCK: flow cytometry clustering without K
FSC: forward scatter
GI: Granularity Index
GPA: granulomatosis with polyangiitis
ImmPort: Immunology Database and Analysis Portal
ITN: Immune Tolerance Network
LDG: low-density granulocyte
LR: likelihood ratio
MPA: microscopic polyangiitis
MPO: myeloperoxidase
NIAID: National Institute of Allergy and Infectious Diseases
PR3: proteinase 3
RAVE: Rituximab in ANCA-Associated Vasculitis Trial
SSC: side scatter
WBC: white blood cells
