# Supplementary figures and images for: Reanalysis of the Rituximab in ANCA-Associated Vasculitis trial identifies granulocyte subsets as a novel early marker of successful treatment
Source: Arthritis Res Ther. 2015 Sep 21;17(1):262. doi: 10.1186/s13075-015-0778-z (PMC4576403; doi:10.1186/s13075-015-0778-z)

(A)

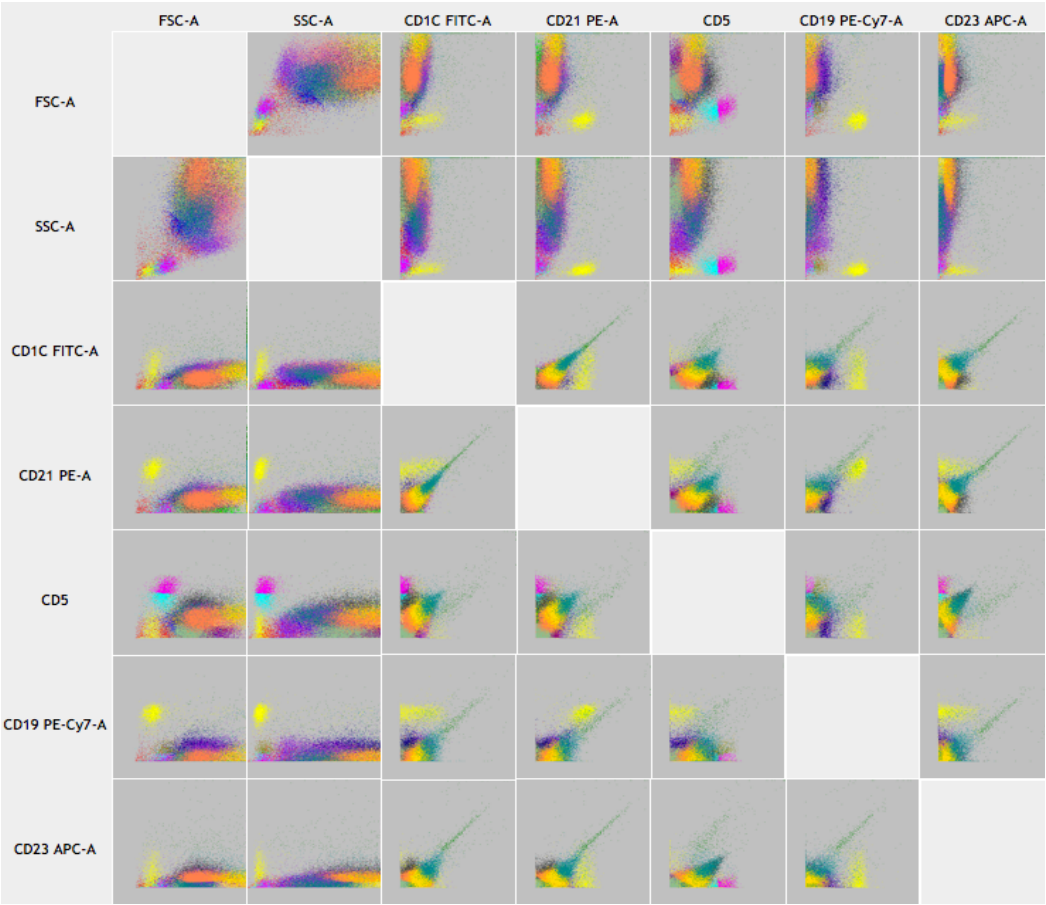

(B)

| ey: 1=negative, 2=low, 3=positive, 4=high |       |       |       |          |          |     |          |         |              |
|-------------------------------------------|-------|-------|-------|----------|----------|-----|----------|---------|--------------|
| op.                                       | Color | FSC-A | SSC-A | CD1C ... | CD21 ... | CD5 | CD19 ... | CD23... | Proportion % |
|                                           |       | 2     | 1     | 1        | 1        | 1   | 1        | 1       | 2.29         |
|                                           |       | 1     | 1     | 1        | 2        | 1   | 2        | 1       | 1.56         |
|                                           |       | 4     | 4     | 2        | 2        | 2   | 2        | 2       | 0.31         |
|                                           |       | 2     | 3     | 1        | 1        | 1   | 1        | 1       | 8.11         |
|                                           |       | 3     | 3     | 1        | 1        | 1   | 1        | 1       | 8.28         |
|                                           |       | 3     | 2     | 1        | 1        | 1   | 1        | 1       | 4.43         |
|                                           |       | 2     | 1     | 1        | 1        | 2   | 1        | 1       | 0.38         |
|                                           |       | 1     | 1     | 1        | 1        | 2   | 1        | 1       | 1.09         |
|                                           |       | 2     | 1     | 1        | 1        | 2   | 1        | 1       | 1.02         |
| 0                                         |       | 3     | 4     | 1        | 1        | 1   | 1        | 1       | 7.62         |
| 1                                         |       | 3     | 4     | 1        | 1        | 1   | 1        | 1       | 5.0          |
| 2                                         |       | 4     | 3     | 1        | 1        | 1   | 1        | 1       | 5.86         |
| 3                                         |       | 3     | 4     | 1        | 1        | 1   | 1        | 1       | 8.17         |
| 4                                         |       | 3     | 4     | 1        | 1        | 1   | 1        | 1       | 8.9          |
| 5                                         |       | 2     | 3     | 1        | 1        | 1   | 1        | 1       | 8.07         |
| 6                                         |       | 3     | 4     | 1        | 1        | 2   | 1        | 1       | 3.23         |
| 7                                         |       | 3     | 3     | 1        | 1        | 1   | 1        | 1       | 6.47         |
| 8                                         |       | 3     | 3     | 1        | 1        | 1   | 1        | 1       | 3.09         |
| 9                                         |       | 3     | 4     | 1        | 1        | 1   | 1        | 1       | 8.2          |
| 0                                         |       | 4     | 4     | 1        | 1        | 1   | 1        | 1       | 5.8          |
| 1                                         |       | 4     | 4     | 2        | 2        | 2   | 1        | 1       | 2.14         |

(C)

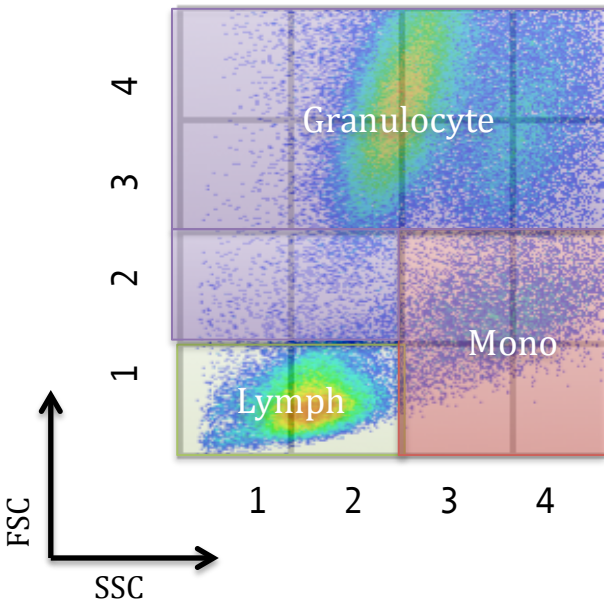

Supplement: Additional file 1: — ImmPort-FLOCK Output. Sample FLOCK analysis of an FCS file (903935_SN05_I019.fcs). Distinct populations identified by clustering are color-coded. a Dot plot representations of populations defined by FSC, SSC, CD1c, CD21, CD5, CD19 and CD23. b Summary of each population described by an expression profile of approximate marker intensity (1 = negative, 2 = low, 3 = positive, 4 = high). c Gridding definitions used to identify total lymphocyte, monocyte and granulocyte populations on the basis of FSC and SSC. (PDF 643 kb) [file 13075_2015_778_MOESM1_ESM.pdf]

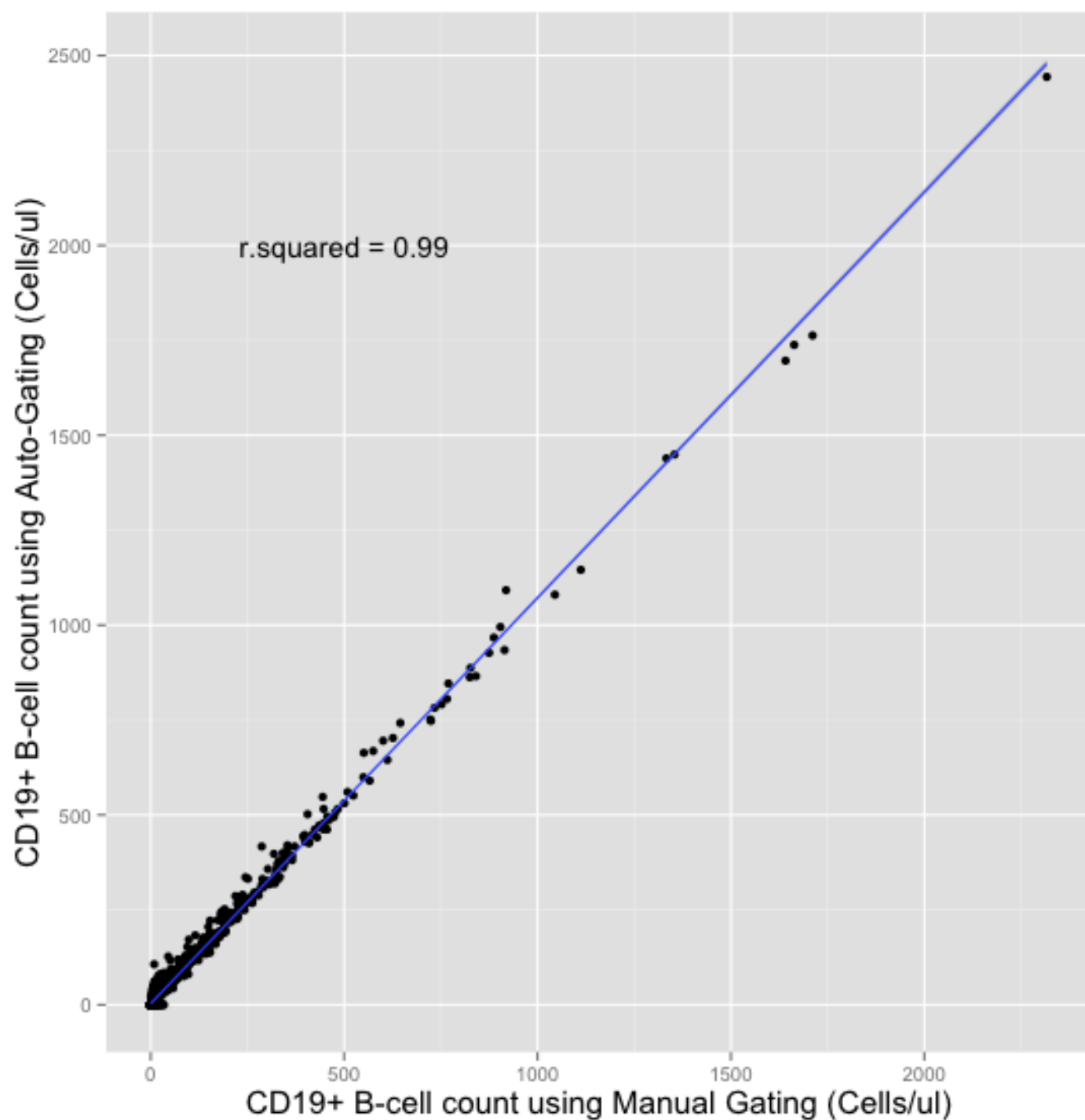

Supplement: Additional file 2: — Correlation between CD19+ B cells identified manually versus via ImmPort-FLOCK. RAVE flow cytometry files were analyzed using ImmPort-FLOCK, and the population of CD19+ lymphocytes was identified for each of the 1150 files. Publicly available manual gating results from primary RAVE trial investigators (x-axis) were compared with ImmPort-FLOCK results (y-axis). A regression coefficient was calculated using a linear regression model. (PDF 49 kb) [file 13075_2015_778_MOESM2_ESM.pdf]
